# Differential miRNA Expression in Cells and Matrix Vesicles in Vascular Smooth Muscle Cells from Rats with Kidney Disease

**DOI:** 10.1371/journal.pone.0131589

**Published:** 2015-06-26

**Authors:** Praneet Chaturvedi, Neal X. Chen, Kalisha O’Neill, Jeanette N. McClintick, Sharon M. Moe, Sarath Chandra Janga

**Affiliations:** 1 Department of Biohealth Informatics, School of Informatics and Computing, Indiana University Purdue University, Indianapolis, Indiana, United States of America; 2 Division of Nephrology, Department of Medicine, School of Medicine, Indiana University, Indianapolis, Indiana, United States of America; 3 Roudebush VA Medical Center, Indianapolis, Indiana, United States of America; 4 Center for Computational Biology and Bioinformatics, Indiana University School of Medicine, 5021 Health Information and Translational Sciences (HITS), Indianapolis, Indiana, United States of America; 5 Department of Medical and Molecular Genetics, Indiana University School of Medicine, Medical Research and Library Building, Indianapolis, Indiana, United States of America; University of Massachusetts Medical, UNITED STATES

## Abstract

Vascular calcification is a complex process and has been associated with aging, diabetes, chronic kidney disease (CKD). Although there have been several studies that examine the role of miRNAs (miRs) in bone osteogenesis, little is known about the role of miRs in vascular calcification and their role in the pathogenesis of vascular abnormalities. Matrix vesicles (MV) are known to play in important role in initiating vascular smooth muscle cell (VSMC) calcification. In the present study, we performed miRNA microarray analysis to identify the dysregulated miRs between MV and VSMC derived from CKD rats to understand the role of post-transcriptional regulatory networks governed by these miRNAs in vascular calcification and to uncover the differential miRNA content of MV. The percentage of miRNA to total RNA was increased in MV compared to VSMC. Comparison of expression profiles of miRNA by microarray demonstrated 33 miRs to be differentially expressed with the majority (~ 57%) of them down-regulated. Target genes controlled by differentially expressed miRNAs were identified utilizing two different complementary computational approaches Miranda and Targetscan to understand the functions and pathways that may be affected due to the production of MV from calcifying VSMC thereby contributing to the regulation of genes by miRs. We found several processes including vascular smooth muscle contraction, response to hypoxia and regulation of muscle cell differentiation to be enriched. Signaling pathways identified included MAP-kinase and wnt signaling that have previously been shown to be important in vascular calcification. In conclusion, our results demonstrate that miRs are concentrated in MV from calcifying VSMC, and that important functions and pathways are affected by the miRs dysregulation between calcifying VSMC and the MV they produce. This suggests that miRs may play a very important regulatory role in vascular calcification in CKD by controlling an extensive network of post-transcriptional targets.

## Introduction

Vascular (arterial) calcification is a prominent finding in aging, diabetes, chronic kidney disease (CKD), and inflammatory diseases, and is associated with increased morbidity and mortality [1]. The pathophysiology of vascular calcification (also called mineralization) is complex, but appears to be similar to normal bone osteogenesis. Vascular smooth muscle cells (VSMC) alter to an osteoblast like phenotype which synthesizes matrix vesicles (MV), 50–200 nm spheres that contain calcium and phosphate and initiate mineralization within the extracellular matrix. MVs are present by electron microscopy in normally calcified tissues such as bone and cartilage and in aberrant calcification sites such as vascular calcification. These osteoblast like cells also secrete the extracellular matrix (ECM) proteins on which MVs initiate mineralization [2, 3]. The protein content of MV appears to be a central determinant of the ability to mineralize the ECM. We have previously demonstrated that MV with increased annexin II and VI and minimal fetuin-A content can mineralize on an ECM, but MV with decreased content of annexin II and VI and increased fetuin-A do not [4].

Matrix vesicles are similar to microparticles and exosomes, and studies have demonstrated the ability of these other vesicle like structures from non-mineralizing cells to transfer RNA or microRNA (miRNA) to new cells [5] facilitating cell to cell or cell to extracellular matrix communication. MiRNA are fragments of noncoding RNA typically ~22 nucleotides in length that negatively regulate protein-coding genes by acting as posttranscriptional repressors of gene expression. MicroRNAs suppress gene expression via imperfect base pairing to the 3’ untranslated region (3’ UTR) of target mRNAs leading to repression of protein production or mRNA degradation. Accumulating evidence indicates that miRNA’s play a crucial role in regulating various cellular processes by controlling the expression of vast number of genes post-transcriptionally [6]. Importantly, a single miRNA may affect transcription of multiple genes involved in common pathways.

Computational methods have played an important role in the prediction of miRNA targets from the very beginning. Traditionally, some major features such as hairpin-shaped stem loop structure, high minimal folding free-energy, gene expression relationships between miRNA-mRNA pairs and high evolutionary conservation of the seed regions have been used in the computational identification of targets [6]. In this study, we have therefore employed two different complementary computational approaches which employ these features, namely Miranda [4] and Targetscan [7] to predict the mRNA targets of the differentially expressed miRNAs. In the current study, we aimed to understand the differentially present miRNA in calcifying VSMC (incubated with high phosphorus media) and the MV they produce. We used miRNA array and bioinformatics analyses to identify dysregulated miRNA in CKD animals that may provide insight into the cellular regulation of MV packaging of miRNA and to determine what post-transcriptional networks are involved as VSMC initiate calcification.

## Materials and Methods

### Animal models and cell culture

Primary rat vascular smooth muscle cells (VSMC) were isolated from our rodent model of Chronic Kidney Disease-Mineral Bone Disorder (CKD-MBD), the Cy/+ rat model cystic kidney disease (CKD rat). This model spontaneously develops all three manifestations of CKD-MBD: biochemical abnormalities, extraskeletal calcification, and abnormal bone [8]. Briefly, VSMC were isolated from the descending thoracic aorta in CKD rats (age 35 week old) by the explant method as previously described [7] and grown in Dulbecco’s Modified Eagles Medium (DMEM; Sigma, St. Louis, MO), with 10% FBS. To induce calcification, VSMC were treated with 5 mmol/L β-glycerophosphate, 1 U/ml fetal alkaline phosphatase, 10^-7^ mol/L insulin and 50 μg/ml ascorbic acid in the presence of 15% serum [9]. The β-glycerophosphate is cleaved by the alkaline phosphatase to release phosphorus and thus this approach allows VSMC to be incubated in high phosphorus media without concerns of spontaneous precipitation. Such high phosphorus “calcifying” media induced the de-differentiation needed for VSMC to calcify in vitro [4, 9]. All procedures were reviewed and approved by the Indiana University School of Medicine Institutional Animal Care and Use Committee.

### Matrix vesicle (MV) isolation

MV were isolated from calcifying VSMC obtained from three CKD rats by collagenase digestion as previously described [4]. Calcifying VSMC were incubated with crude collagenase (500 U/ml, type IA, Sigma) in a solution of 0.25 M sucrose, 0.12 M NaCl, 0.01 M KCL and 0.02 M Tris buffer, pH 7.45, at 37°C for 3 hrs. The digests were centrifuged at 800 g and 30,000 g to remove cell debris and microsomes, respectively. The supernatant was centrifuged at 250,000 g to pellet the MV followed by resuspension in TBS (pH 7.6) with 0.25 M sucrose. The MV amount was determined by protein concentration (Bio-Rad). We used 3 different CKD rats to isolate 3 different VSMC cultures. Each of these three cultures was used to isolated MV. We then ran separate arrays on each of the VSMC-MV pairs.

### RNA isolation, quantification and micro-RNA array

Total RNA from VSMC or MV was isolated using miRNeasy Mini Kit (Qiagen) according to the manufacturer’s instructions. Total RNA was eluted from the column in RNase-free water and stored at -80°C. Quantification of miRNA was performed at the Center for Genetics in Indiana University School of Medicine using Agilent 2100 Bioanalyzer Small RNA kit.

To perform the arrays, total RNA samples were labeled using the Genisphere FlashTag HSR kit. The labeled samples were individually hybridized to Affymetrix GeneChip miRNA 3.0 arrays. They were stained and washed using the standard miRNA protocol. Affymetrix GeneChip Command Console Software (AGCC) was used to scan the arrays and generate CEL files. CEL files were imported into Partek Genomics Suite (Partek, Inc., St. Louis, Mo). RMA (robust multi-array average) signals were generated for all probe sets using the RMA background correction, quantile normalization and summarization by Median Polish [10]. Summarized signals for each probe set were log_2_ transformed. These log transformed signals were used for Principal Components Analysis, hierarchical clustering and signal histograms to determine if there were any outlier arrays. No outliers were detected in this analysis. Untransformed RMA signals were used for fold change calculations. Contrasts were calculated as required. Fold changes were calculated using the untransformed RMA signals. Probe sets with log_2_ expression levels < 1.0 were considered very close to background. Probe sets with average expression levels < 1.0 were removed before the False Discovery Rate (FDR) was calculated using the Storey method [11].

### Identification of dysregulated miRNAs in MV vs VSMC in CKD rats

**Fig 1:**
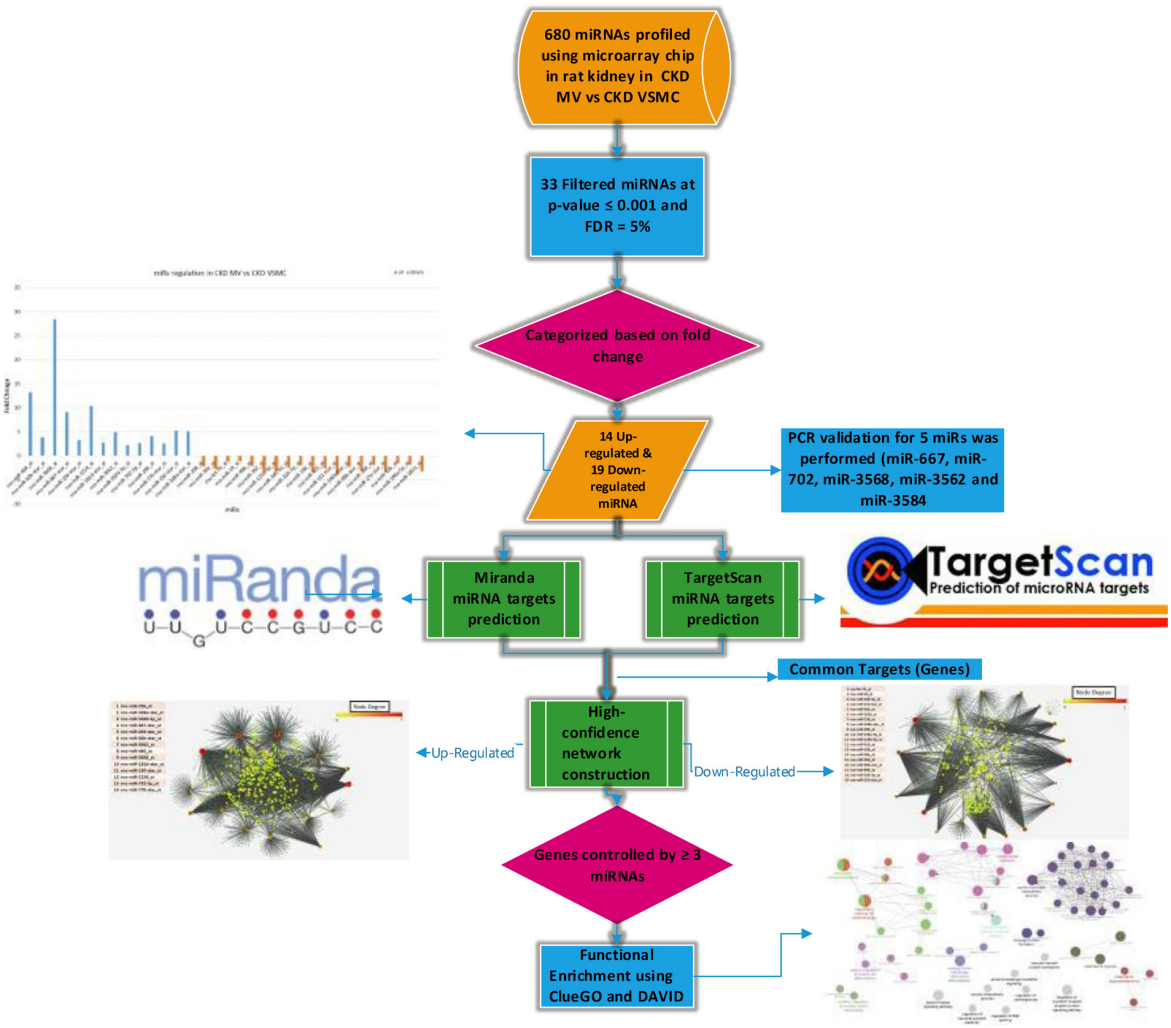
Flowchart showing the various steps involved in this study. Flowchart summarizing the sequence of analyses performed in this study.

The sequence of analyses performed in this study was shown in flowchart in Fig 1. We had a dataset of 680 rat miRNA’s profiled experimentally in the chronic kidney disease rats in both Matrix Vesicles (MV) and Vascular Smooth Muscle Cells (VSMC). Their expression change was measured in terms of fold change in expression from MV to VSMC in these CKD rats. We used highly stringent thresholds of p-value < = 0.001, FDR = 5% to obtain a set of dysregulated miRs between these two conditions.

### Target prediction for miRNAs

For predicting miRNA targets in the rat genome, we used two different approaches which employ complementary criteria, in order to obtain highest prediction power and reliability. These methods included 1) Miranda, which is a machine learning method for ranking microRNA target sites by a down-regulation score by integrating gene expression and sequence level features. The algorithm trains a regression model on sequence and contextual features extracted from high confidence miRanda-predicted target sites and hence can predict non-canonical and non-conserved sites producing complementary predictive power to other existing approaches [12] and 2) Targetscan: predicts targets for miRNA’s using context scores based on the extent of conservation of the seed region in the target mRNAs 3’ UTR across various mammalian orthologs [13]. For Miranda, we obtained 3’UTR fasta sequence file from biomart-ensembl (http://www.ensembl.org/biomart/martview) for the rat genome and used it as the input. For Targetscan, we used the 3’UTR multiple sequence alignment file available from the target scan database for rattus norvegicus. To predict the mRNA targets for the set of dysregulated miRs, for Miranda we used score(S) > 150 and free energy (ΔG) < -22kcal/mol as the thresholds while for Targetscan we used context+score ≤ -0.3 for identifying genes post-transcriptionally controlled by the miRs. Complete set of targets predicted by both the methods for the dysregulated miRs are shown in S1 Table.

### Constructing high-confidence post-transcriptional regulatory network of dysregulated miRs

Most microRNAs control the expression of hundreds of target transcripts in a cell, typically by base-pairing with the 3’ untranslated regions, resulting in a global post-transcriptional regulatory network [6]. To construct a high-confidence post-transcriptional regulatory network of miRNAs which are dysregulated between VSMC and MV in CKD rats, we first identified the target transcripts of miRNAs using two different well-established computational methods for miRNA target prediction, namely Miranda and Targetscan, as described above. Resulting predictions were then filtered to include only those mRNAs identified as targets of each miRNA, by both the computational methods—referred to as the intersection approach, thus allowing the construction of a high confidence network. In other words, we used predictions from Miranda and TargetScan and confirmed a target for miR only if it is predicted by both the methods at default thresholds. Using this approach we constructed a network of miR and its targets for all dysregulated miRs. Though this approach reduces the number of targets for miRs, it ensures to generate a robust set of miR targets allowing the analysis and interpretation of the miRNA controlling network in vascular calcification.

### Constructing a network of genes controlled by multiple miRs

We obtained the genes which are controlled by at least 3 dysregulated miRs and then grouped these networks into two sections 1) Genes controlled by at least 3 miRs which are enriched in MV compared to VSMC and 2) Genes controlled by at least 3 depleted miRs in MV compared to VSMC. Both of these networks were derived from high-confidence network constructed in the previous step. Further, we performed functional enrichment of genes in these networks to identify the pathways and functions affected due to the dysregulation of miRNAs.

### Functional enrichment of genes controlled by multiple miRs

Since miRNA-mediated gene expression change can contribute to functional and phenotypic consequences, we identified the genes controlled by at least 3 dysregulated miRNAs to uncover the functions and pathways which are affected due to their dysregulation. Genes which were controlled by at least 3 miRs were used for functional enrichment analysis using ClueGO—a plugin in cytoscape which helps in the visualization of the enriched biological functions in terms of a network [14]. We also used David [15, 16] a functional/pathway enrichment tool to independently compare our functional analysis results. This analysis allowed us to predict the pathways and functions which are likely disrupted or altered due to variations in the regulation at post-transcriptional level by miRs.

### Assignment of functional roles to miRs

We also obtained enriched functions in the targets predicted to be controlled by miRs, by performing a functional enrichment of the genes controlled by miRs individually. We used David [15, 16] to extract enriched functions for each miR. All the functions obtained were filtered at p-value < = 0.01 (FDR < 5%) and only those miRs which had at least one significant function are shown in Table 1.

**Table 1:** Enriched functions associated with dysregulated miRs based on the functional enrichment of the predicted targets.

miR	Enriched Functions	Supporting Publications
**rno-miR-299_st (↓)**	**phosphoprotein**	**NA**
**rno-miR-30e_st (↓)**	**prenylation, mutagenesis site**	**[24]**
**rno-let-7b_st (↓)**	**GO:0000166~nucleotide binding, GO:0001882~nucleoside binding**	**NA**
**rno-miR-24_st (↓)**	**phosphoprotein, GO:0046983~protein dimerization activity, GO:0008610~lipid biosynthetic process**	**[28]**
**rno-miR-99b_st (↓)**	**Malaria**	**NA**
**rno-miR-324-3p_st (↓)**	**phosphotransferase, GO:0030005~cellular di-, tri-valent inorganic cation homeostasis, GO:0007243~protein kinase cascade, alternative splicing, GO:0006468~protein amino acid phosphorylation, GO:0001754~eye photoreceptor cell differentiation, GO:0007242~intracellular signaling cascade**	**NA**
**rno-miR-19b_st (↓)**	**phosphoprotein, GO:0006163~purine nucleotide metabolic process, alternative splicing, GO:0007264~small GTPase mediated signal transduction, prenylation, GO:0006164~purine nucleotide biosynthetic process**	**NA**
**rno-miR-423_st (↓)**	**nucleotide-binding, GO:0007242~intracellular signaling cascade**	**NA**
**rno-miR-106b-star_st (↓)**	**GO:0007264~small GTPase mediated signal transduction, IPR005225:Small GTP-binding protein**	**NA**
**rno-miR-99b-star_st (↓)**	**mutagenesis site**	**NA**
**rno-miR-30d_st (↓)**	**prenylation**	**[24]**
**rno-miR-27a-star_st (↓)**	**GO:0007242~intracellular signaling cascade**	**[29]**
**rno-miR-199a-5p_st (↓)**	**Signal-anchor, phosphoprotein, GO:0006631~fatty acid metabolic process, IPR018503:Tetraspanin, conserved site**	**NA**
**rno-miR-181b_st (↓)**	**GO:0010608~posttranscriptional regulation of gene expression**	**[30]**
**rno-miR-92b-star_st (↑)**	**GO:0043009~chordate embryonic development, GO:0006814~sodium ion transport, GO:0044057~regulation of system process**	**[31]**
**rno-miR-3568_st (↑)**	**GO:0007188~G-protein signaling, coupled to cAMP nucleotide second messenger**	**NA**
**rno-miR-667-star_st (↑)**	**alternative splicing, GO:0016192~vesicle-mediated transport, GO:0007158~neuron adhesion**	**NA**
**rno-miR-204-star_st (↑)**	**GO:0032268~regulation of cellular protein metabolic process, GO:0006468~protein amino acid phosphorylation, GO:0008284~positive regulation of cell proliferation, GO:0010604~positive regulation of macromolecule metabolic process, GO:0044093~positive regulation of molecular function, GO:0005343~organic acid:sodium symporter activity**	**[27]**
**rno-miR-1224_st (↑)**	**cell cycle control, hsa04310:Wnt signaling pathway**	**NA**
**rno-miR-181d-star_st (↑)**	**GO:0008289~lipid binding, GO:0006811~ion transport**	**[30]**
**rno-miR-3562_st (↑)**	**GO:0048640~negative regulation of developmental growth, GO:0010324~membrane invagination, GO:0006897~endocytosis, GO:0022898~regulation of transmembrane transporter activity, GO:0016192~vesicle-mediated transport, GO:0006811~ion transport, GO:0010608~posttranscriptional regulation of gene expression**	**NA**
**rno-miR-3584-5p_st (↑)**	**mutagenesis site, GO:0001609~adenosine receptor activity, G-protein coupled, GO:0010604~positive regulation of macromolecule metabolic process, alternative splicing**	**NA**
**rno-miR-702-5p_st (↑)**	**disease mutation**	**NA**
**rno-miR-296_st (↑)**	**hsa04270:Vascular smooth muscle contraction**	**NA**
**rno-miR-770-star_st (↑)**	**nucleotide-binding**	**NA**
**rno-miR-150-star_st (↑)**	**GO:0004842~ubiquitin-protein ligase activity, GO:0043632~modification-dependent macromolecule catabolic process,**	**NA**
**rno-miR-328a-star_st (↑)**	**GO:0030182~neuron differentiation, GO:0004672~protein kinase activity, GO:0016486~peptide hormone processing, GO:0031668~cellular response to extracellular stimulus, GO:0006915~apoptosis, GO:0014070~response to organic cyclic substance, GO:0031012~extracellular matrix, GO:0009967~positive regulation of signal transduction, GO:0008284~positive regulation of cell proliferation, GO:0035270~endocrine system development, GO:0010647~positive regulation of cell communication, GO:0048168~regulation of neuronal synaptic plasticity**	**NA**

All the functions shown are significant at p-value < = 0.01. MicroRNAs whose levels are higher in MV compared VSMC are highlighted with an upward arrow **(↑)** in the first column, while those which were found to occur in lower levels in MV are suffixed with a downward arrow **(↓).**

### Confirmation by real-time PCR

We then confirmed miRNA expression of selected miRNA that controlled multiple genes and/or controlled pathways which are known to be important in vascular calcification, by real time PCR as previously described [17] using TaqMan miRNA assays (Applied Biosystems, Foster City, CA). Forty ng of total RNA were used for reverse transcription to synthesize complementary DNA using TaqMan miRNA-specific primers and the Taq reverse transcription kit (Applied Biosystems, Foster City, CA). Target-specific PCR primers (miR-667, miR-702, miR-3562, miR-3568 and miR-3584) were obtained from Applied Biosystems. Real-time PCR amplifications were performed using TaqMan miRNA Assays (TaqMan MGP probes, FAM dye-labeled) using Applied Biosystems 7500 Real-Time PCR systems (Applied Biosystems). The cycle number at which the amplification plot crosses the threshold was calculated (C_T_), and the ΔΔC_T_ method was used to analyze the relative changes in gene expression and normalized by U6, a non-human ubiquitous miRNA.

## Results

### MV contains greater amount of miRNA but less total RNA compared to VSMC

**Fig 2:**
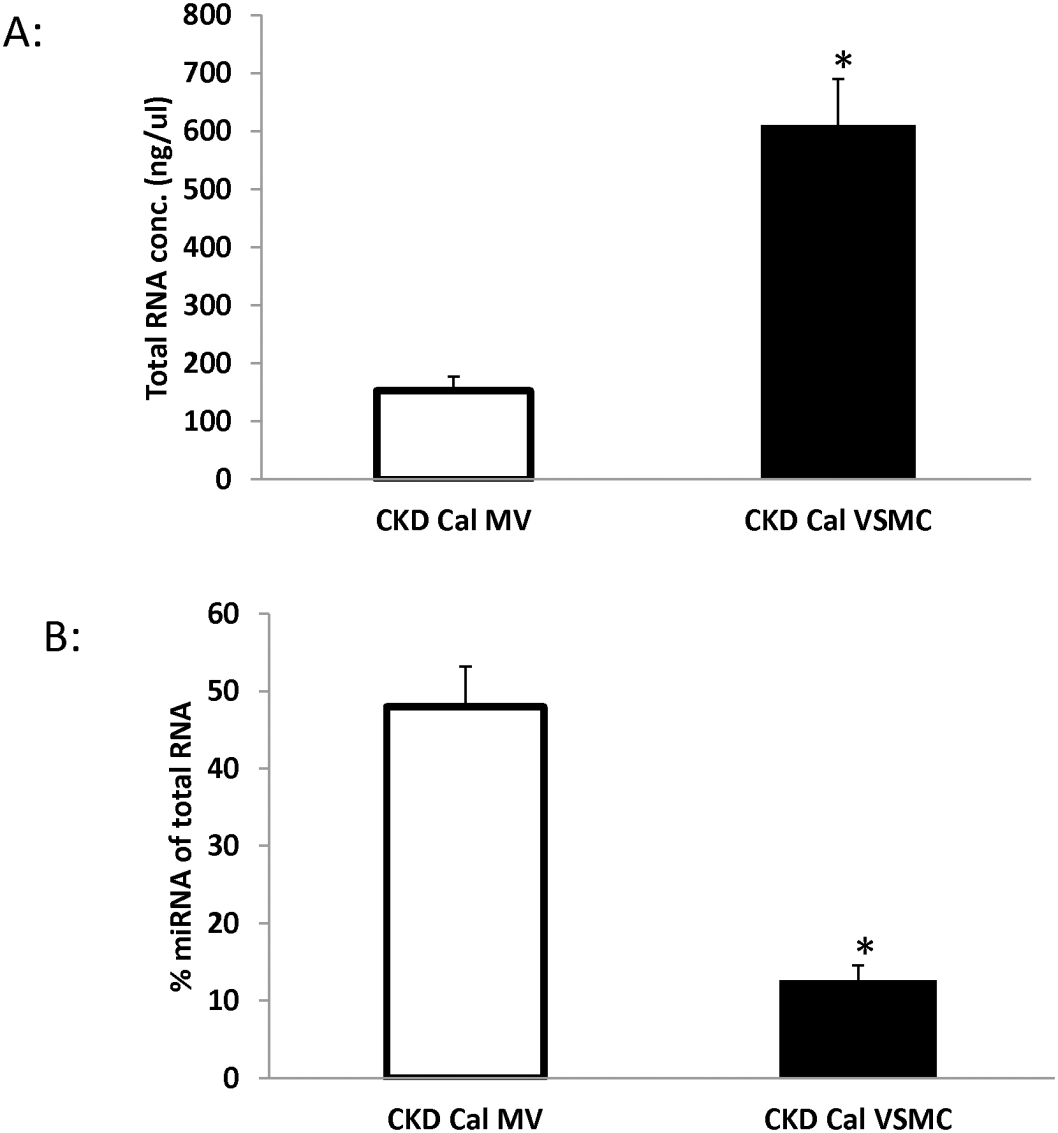
miRNA is concentrated in MV compared to VSMC from CKD rats. Total RNA from VSMC or MV was isolated from CKD rats and quantification performed using Agilent 2100 Bioanalyzer total and Small RNA kit. The results demonstrated that the total RNA level (A) is greater but miRNA levels are lower in VSMC compared to MV (B). n = 3, Data were expressed as mean±SEM, *p<0.05, MV vs. VSMC.

To determine the total RNA and miRNA concentration in MV compared to VSMC which they are originated, total RNA was isolated from MV and VSMC and the relative concentrations of total RNA and miRNA determined by Agilent 2100 Bioanalyzer total RNA and Small RNA kits, respectively. The results demonstrated that total RNA concentration is 4 times greater in VSMC than that in MV (Fig 2A). In contrast, percent of miRNA of total RNA in MV is 4 times greater than that in VSMC (Fig 2B), confirming that miRNA are concentrated in MV as they are in exosomes.

### Several genes were found to be controlled by dysregulated miRs

**Fig 3:**
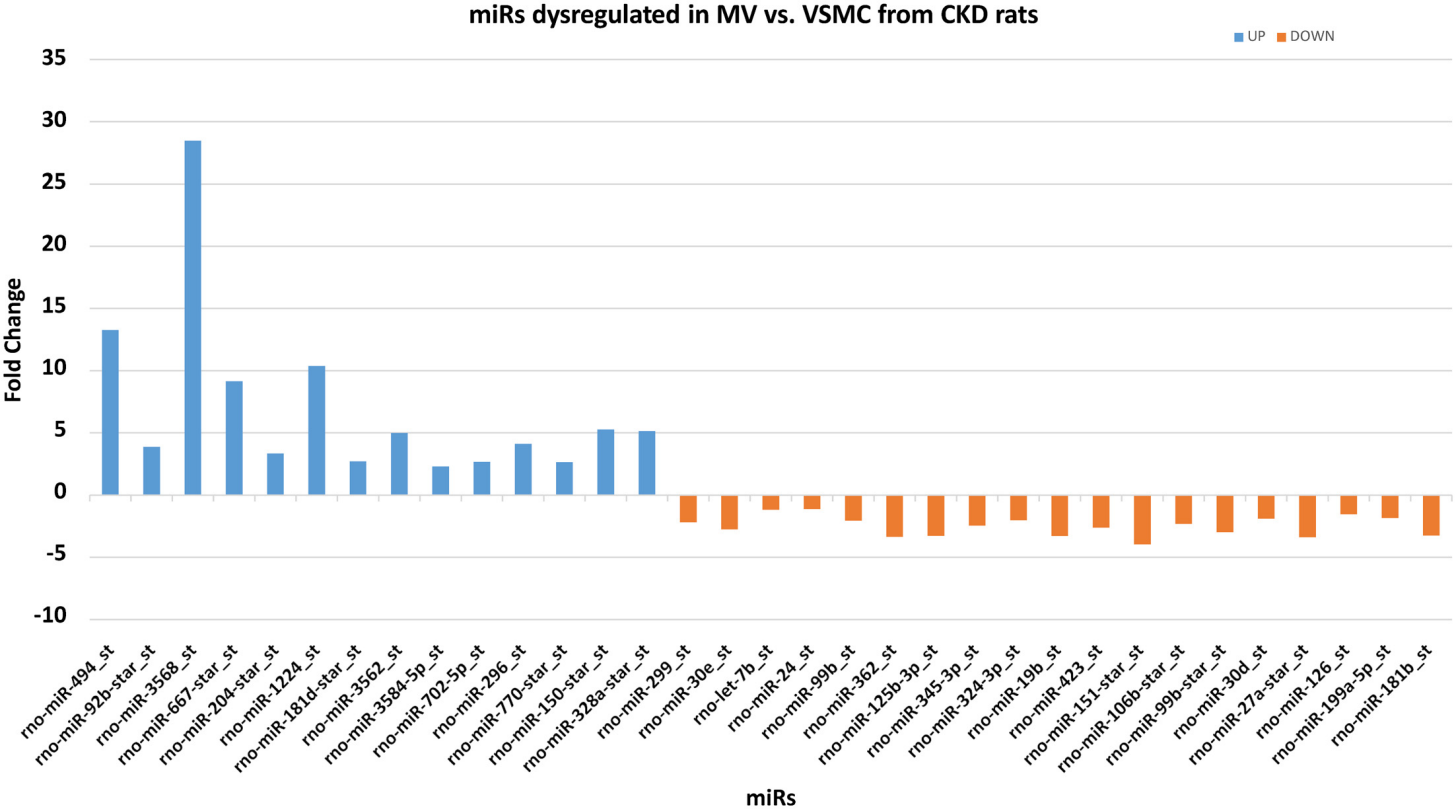
The fold change in expression of 33 miRs dysregulated in MV compared to VSMC from CKD rats. All the 33 miRs were filtered at p-value < = 0.001 and FDR = 5% and isolated from 3 different rats Blue color represents the up-regulated miRs whereas the orange color represents the down-regulated miRs.

**Fig 4:**
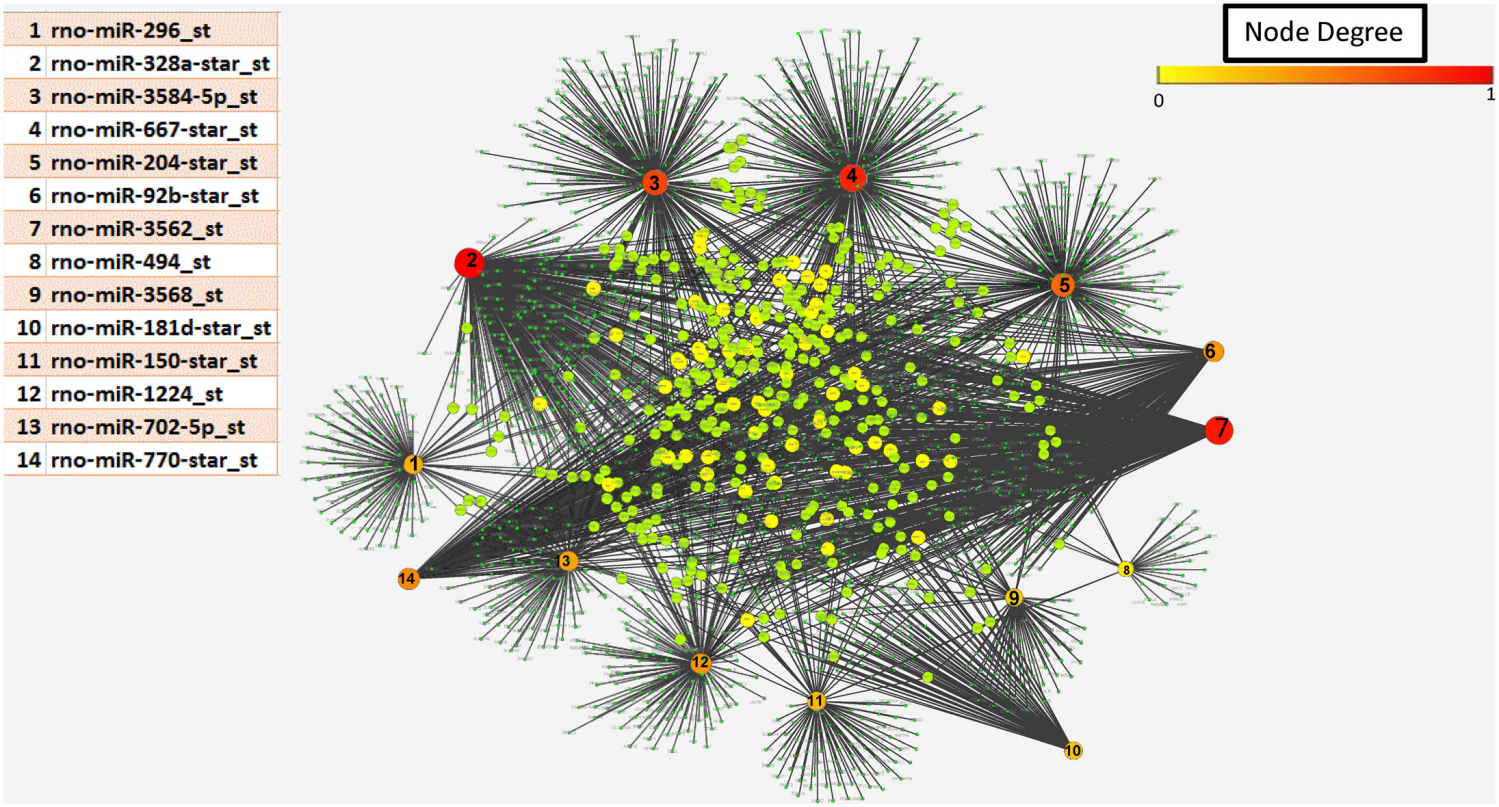
Network of up-regulated miRs and the targets (genes) controlled by them. Red/orange nodes represent miRNA, with number corresponding to the table; the larger the node the more genes targeted. The green/yellow nodes represent genes regulated by these miRNA and the black lines the connectivity between miRNa and target genese. The highest degree which was observed to be 381 targets in this network. Network is generated using Cytoscape.

**Fig 5:**
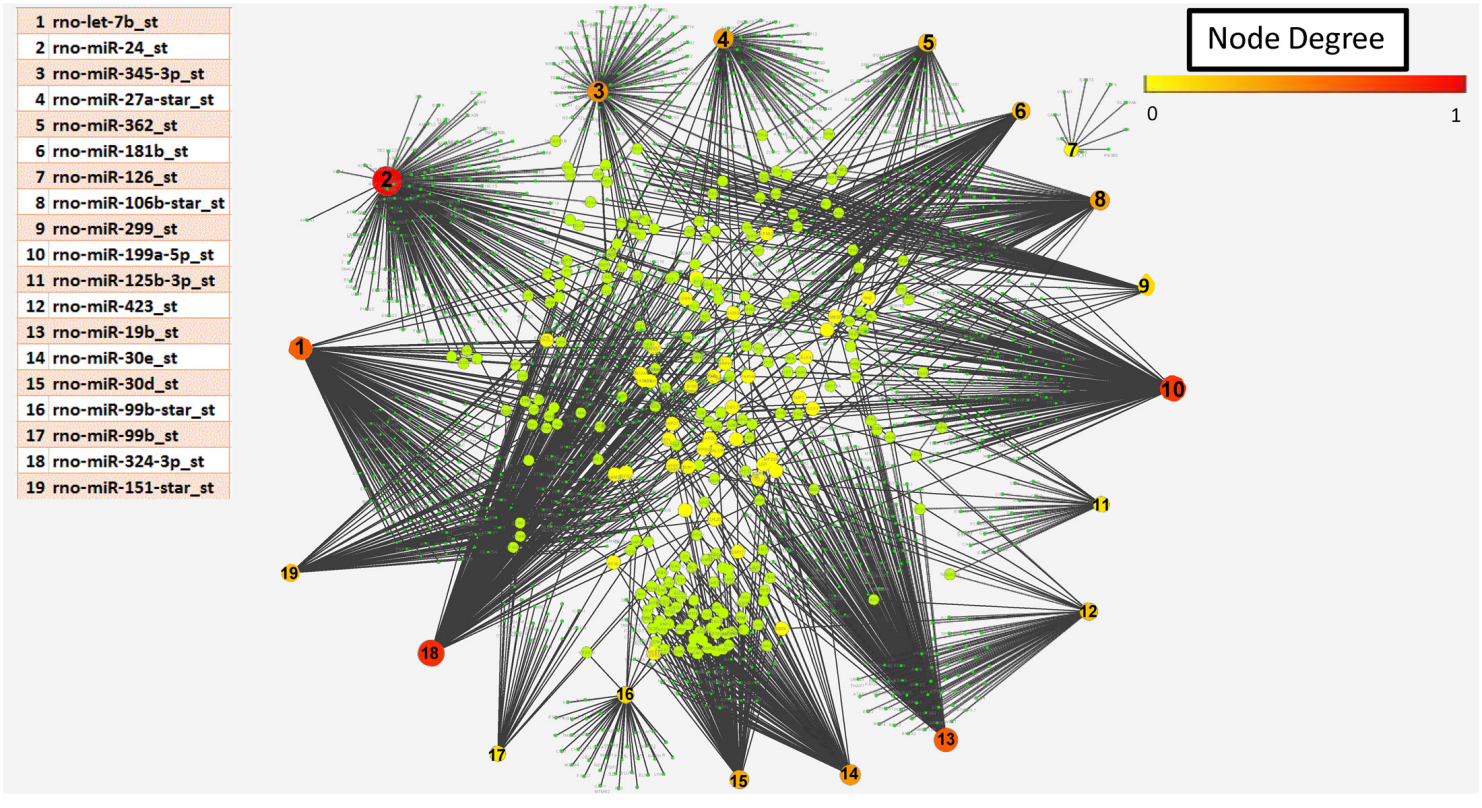
Network of down-regulated miRs and the targets (genes) controlled by them. Color transition of the nodes is based on the degree (number of connections) of the nodes. Here nodes comprise both miRs and their targets. A table in the figure provides information about the nodes corresponding to the miRs. 0 represents lowest degree that is 1 and 1 corresponds to highest degree which was observed to be 262 targets in this network. Red color corresponds to the highest degree and green color corresponds to lowest degree. Network is generated using cytoscape.

Micro RNA array demonstrated that 14 miRNAs increased by 2 fold or more (defined as enriched set of miRNAs) and 19 miRNA decreased at least by 2 fold or more (depleted set) in MV compared to the calcifying VSMC from which they originate using the high stringency criteria (Fig 3). By both the computational methods (Miranda and Targetscan) we demonstrated that in the enriched set, miR-328a-star_st was found to control maximum number of targets (381) followed by miR-3562 controlling 341 putative targets. In the set of depleted miRs, we found miR-24 to control 262 target mRNAs followed by miR-324-3p which controlled 215 targets. We generated network visualizations showing the network for the two sets of miRs controlling their targets using Cytoscape [18]. Fig 4 shows a network comprising of 2572 edges with all their enriched miRs and their controlled target genes. Similarly, a total of 1933 edges comprising of 19 depleted miRs and predicted targets are shown in Fig 5. As is evident from the figure miRs 328a, 3584, 667 and 3562 were found to control a large number of targets among the enriched set of miRs while the dominant players in the depleted set included miRs 24, 199a-5p, 324-3p and 19b. Further, we obtained genes which are controlled by at-least 3 miRs either enriched or depleted. This resulted in a set of 235 genes to be controlled by at least 3 miRs. Table 1 shows the list of all the dysregulated miRs for which functions and processes they control could be confidently predicted (p-value < = 0.01). Functional enrichment was performed using David and all the functions obtained at p-value < = 0.01 and top 25% are displayed (For extended information see S2 Table).

### Functional and pathway enrichment of genes controlled by multiple miRs

**Fig 6:**
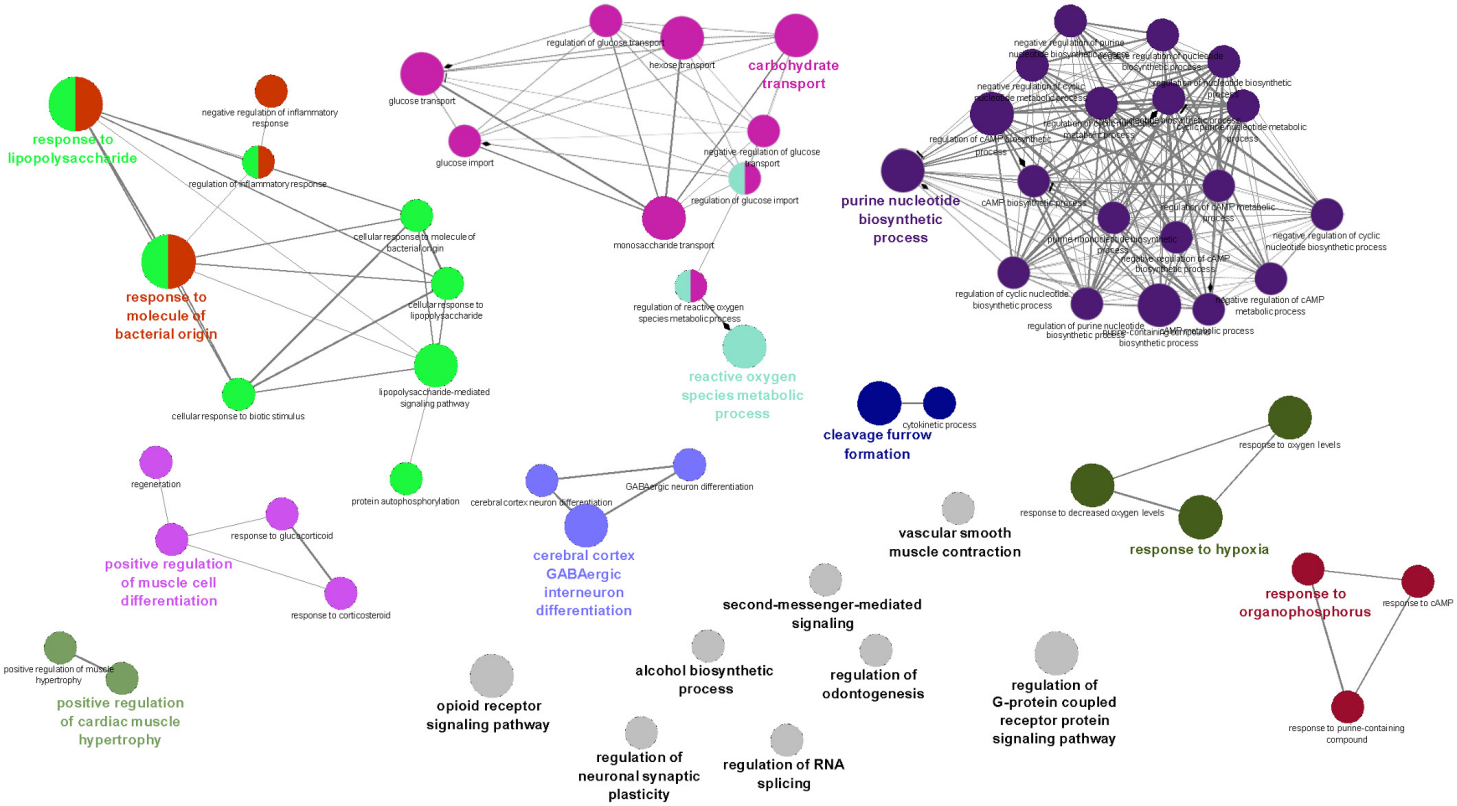
Enrichment of biological functions in the gene set controlled by at least 3 miRs using ClueGo. Analyses of those genese controlled by at least three miRNA were performed to identify important pathways. The genes were categorized based on ClueGo, a plugin of the cytoscape and all the functions reported are at p-value < = 0.05. The size of the nodes represents the number of miRNAs that regulate the genes, and the connectivity of multiple miRNAs regulating multiple genes within a common pathway are shown by the black lines. Purine biosynthesis and cAMP regulation demonstrated the most miRNA regulation (purple nodes).

We performed functional and pathway enrichment for the genes controlled by at least 3 miRs using ClueGo—a plugin in Cytoscape [14] and David—a functional/pathway enrichment tool [15, 16]. We obtained orthologs of genes controlled by at least 3 miRs in mouse as ClueGo is not capable of handling the rat genome and visualized the network of functions obtained at p-value < = 0.05 (Fig 6). Further, we also performed functional and pathway enrichment of the genes using David with the background species set to rat (rattus novergicus) at a p-value < = 0.01 and FDR = 5% (see S1 Fig and S3 Table). The results demonstrated several important pathways and functions to be associated with the genes controlled by multiple dysregulated miRs between VSMC and MV.

### PCR Validation of miRNA expression pattern in MV and VSMC

**Fig 7:**
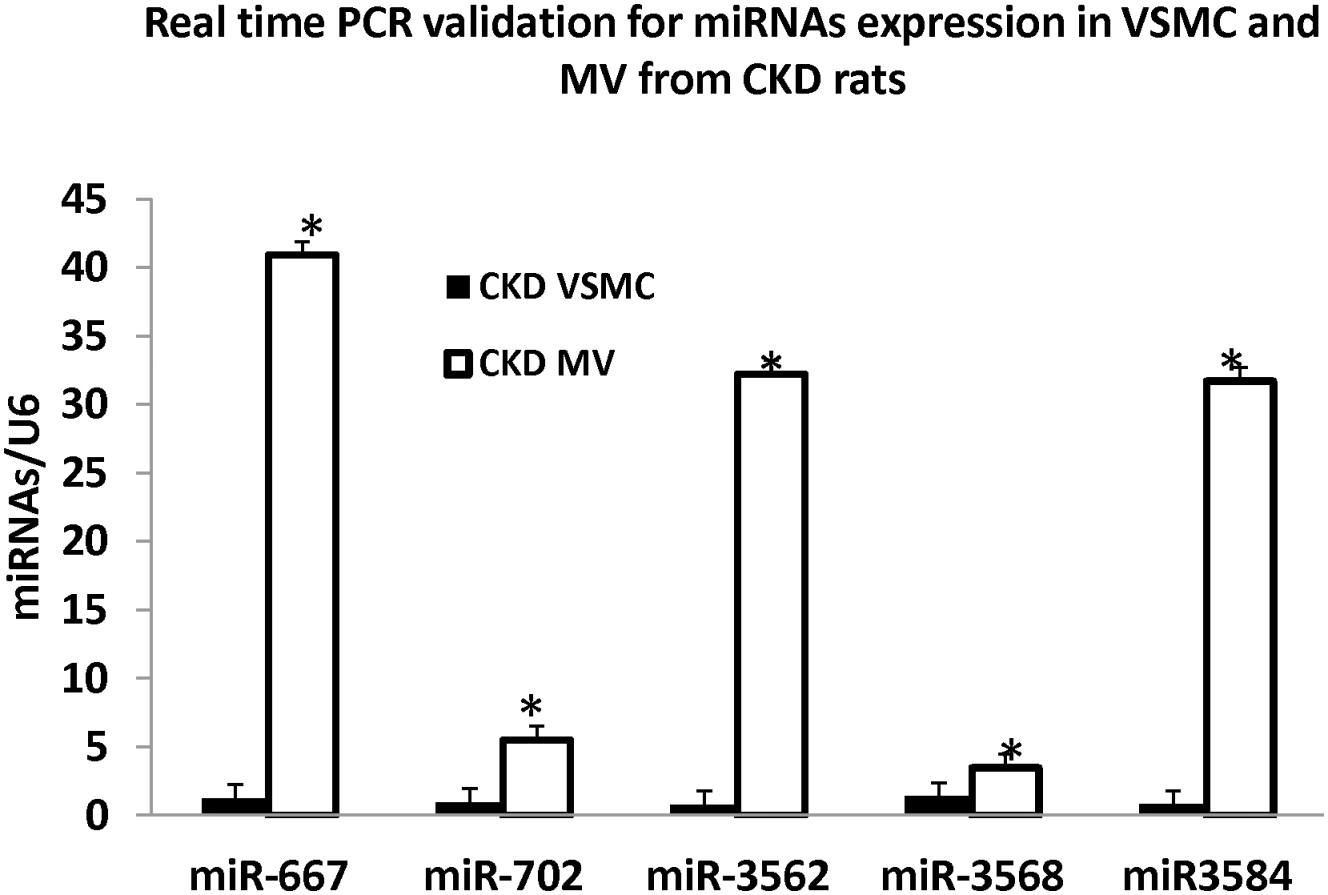
RT-PCR validation of selected miRNAs n MV and VSMC. To validate the miRNA identified by the arrays that regulate multiple genes, we performed Real time PCR on VSMc and MV to determine the expression of miR-667, miR-702, miR-3562, mir-3568 and miR-3584 and normalized by U6. Each sample (n = 3 with MV and VSMC isolated from 3 CKD rats, same samples as arrays) was assayed in triplicate. The results demonstrated increased expression in MV compared to VSMC for each of these miRNAs, confirming the array results. Data were expressed as mean ± SEM. * p<0.05, MV vs. VSMC.

Our microarray analysis and target prediction using computational methods for the 33 miRNAs revealed that the representative set of miRs: miR-667, miR-702, miR-3562, miR-3568 and miR-3584 either controlled genes of interest in the formation of MV, or were predicted to target many genes, with significantly higher expression in MV compared to VSMC from microarray data analysis (Table 1 & Fig 3). Hence, to confirm the expression of these miRNA in MV and VSMC, we performed the real time PCR. The results demonstrated that miR-667, miR-702, miR-3562, miR-3568 and miR-3584 are all expressed in VMSC from CKD rats and are highly concentrated in MV, confirming directional changes from microarray analysis (Fig 7).

## Discussion

VSMC exposed to high levels of phosphorus make MV that calcify an extracellular matrix more than cells not exposed to high phosphorus [4]. In the present study, we compared the expression profiles of VSMC compared to MV from calcifying CKD VSMC cells to identify potential regulatory pathways controlled by miRNAs via the production of MV from calcifying VSMC. As shown in Fig 2, there are 14 miRNAs increased by 2 fold or more and 19 miRNA decreased at least by 2 fold or more in MV compared to VSMC. The arrays were run from MV isolated from VSMC from three different CKD rats, with stringent criteria for selection and thus these miRNA were consistently found altered in MV. These results suggest that the miRNA expression profiles are different in MV compared to the cells from which they originate. Accumulating evidence indicates that miRNA’s play a crucial role in regulating various cellular processes by controlling the expression of vast number of genes post-transcriptionally. MicroRNAs play a critical function during development through effects on cell proliferation, differentiation, and apoptosis [19, 20]. In adults, abnormalities in miRNA expression have been identified in multiple diseases including malignancies, inflammatory and cardiovascular diseases [19, 21].

Several genes can be controlled by multiple miRNAs. To understand the network of miRNA-mRNA associations formed by these miRNAs which are dysregulated, we constructed such a high confident network by including genes which are controlled by multiple dysregulated miRs. We performed functional enrichment of the genes controlled by individual dysregulated miRs to obtain enriched functions for miRs. This enabled the identification and association of most dysregulated miRs with specific functions they are most likely to effect. Several of the predicted functions and processes for these miRs had prior support for their functional roles as is evident from the related citations shown in the table, whereas other pathways had not previously been associated with calcification or exosome production. Key pathways identified include vascular smooth muscle contraction, positive regulation of muscle differentiation, and response to hypoxia. The latter included miRNA that regulate vascular endothelial growth factor (VEGF) a critical regulator of vascular smooth muscle cell function [22]. Our group recently found that VEGF expression may be an indicator of de-differentiation from VSMC cells to osteoblast like cells (unpublished data). Assessment of cell signaling pathways indicated calcium and MAPK signaling pathways to be over-represented in the gene set controlled by dysregulated miRs, MAPK signaling has been shown to be involved in calcification of VMSC [23], but direct confirmation that these miRNA are involved in calcium and MAPK signaling of calcification will require further studies.

Previous studies have shown that miR-30 family members negatively regulate osteoblast differentiation [24] and also suggest RUNX2 and SMAD1 are common post-transcriptional targets of miR-30 family. Our results support and reinforce these observations. Indeed, our high confidence network suggests that RUNX2 is controlled by miR-30d as well as miR-30e and both of these miRs are down-regulated which suggests that they play a major role in regulating osteoblast differentiation. Another recent study suggested that down-regulation of miR-30 family leads to endoplasmic reticulum stress in vascular smooth muscle cells [25] and a similar trend of down-regulation is shown in the case of miR-30d and miR-30e. We also find that ‘response to oxidative stress’ was an enriched pathway reinforcing previous observations that oxidative stress may be involved in both VSMC function and in vascular calcification[26]. In another study by Cui et. al [27], miR-204 has been shown to be involved in regulating vascular smooth muscle cell calcification both in-vivo and in-vitro and our analysis found miR-204 to be up-regulated and controlling several genes which were enriched for regulation of metabolic processes, phosphorylation and cell proliferation. Thus, our results confirm the identification of several miRNAs which were identified in previous studies to be associated with vascular calcification.

Our study also allowed us to uncover several novel miRNAs which have not previously been associated with vascular calcification and/or MV formation. Our results also showed enriched levels of mir-667, mir-702, mir-3562 and mir-3584 as confirmed by RT-PCR data (Fig 7) in MV compared to VSMC. Functional enrichment analysis of these miRs are listed in Table 1 and S2 Table. We confirmed mir-3568, as it was found to be several fold higher in MV compared to VSMC; functional analysis of its ~70 targets revealed enrichment for genes associated with G-protein signaling coupled to cyclic AMP nucleotide second messenger. We examined miR-328a as it also targets many genes that regulate cellular response to extracellular stimulus, extracellular matrix regulation, apoptosis, positive regulation of cell proliferation, and peptide hormone processing. We have previously demonstrated that calcifying MV preferentially mineralize type I, as opposed to type II collagen [4] and thus miRNA involved in extracellular collagen may be important. We also confirmed mir-667 and miR-3562, as these miRNA controlled several genes associated with vesicle-mediated transport and thus may play a role in the formation of MV. The importance of these various pathways in the formation of MV in vascular calcification have not previously been identified and additional validation studies are required. These examples illustrate the power of bioinformatics to identify potential novel pathways. Limitations of the study include the relatively small number of CKD rats studied (n = 3), and the unknown heterogeneity among the rats. Finally, it is unclear if the miRNA results from the CKD rat model accurately reflect miRNA changes in humans with CKD, although miRNA are often evolutionarily conserved. In summary, this study utilized bioinformatics to perform target prediction for dysregulated miRs in MV compared to calcifying CKD VSMC from which they originated. The resulting networks may help unravel the regulatory mechanisms by which miRNA may control vascular calcification via MV released from VSMC. We focused on the complementarity of the target prediction methods, and were very stringent in constructing the network of miRs and their targets. When two different methods predict the same target to be controlled by the miR then it not only validates but helps in reproducibility of results using sub-sampling approach. The identified networks and regulating miRNA provide useful targets for subsequent confirmation studies that may provide novel targets to impede the calcification process in VSMC.

## Supporting Information

S1 Fig Bar-chart representing all the functions enriched for the genes controlled by at-least 3 miRs. This bar-chart provides information on the functions and the number of genes for which the function was enriched at p-value < = 0.05.
(PDF)

S1 Table High confidence predictions of targets for miRs. Miranda and Target scan were used to predict targets for miRs and then high confidence table was designed keeping targets for miRs when predicted by both the methods. Red color represents up-regulated miRs and blue color represents down-regulated miRs.
(XLSX)

S2 Table Top 25% enriched functions of miRs both up-regulated and down-regulated. David was deployed to obtain enriched functions for the genes. All the functions/pathways are enriched at p-value < = 0.01. Blue tab represents down-regulated miR while red tab represents up-regulated miR.
(XLSX)

S3 Table Enriched functions/Pathways for genes controlled by at-least 3 miRs. David was deployed to obtain enriched functions for the genes. All the functions/pathways are enriched at p-value < = 0.01 and FDR = 5%.
(XLSX)
