# Supplementary figures and images for: Differential miRNA Expression in Cells and Matrix Vesicles in Vascular Smooth Muscle Cells from Rats with Kidney Disease
Source: PLoS One. 2015 Jun 26;10(6):e0131589. doi: 10.1371/journal.pone.0131589 (PMC4482652; doi:10.1371/journal.pone.0131589)

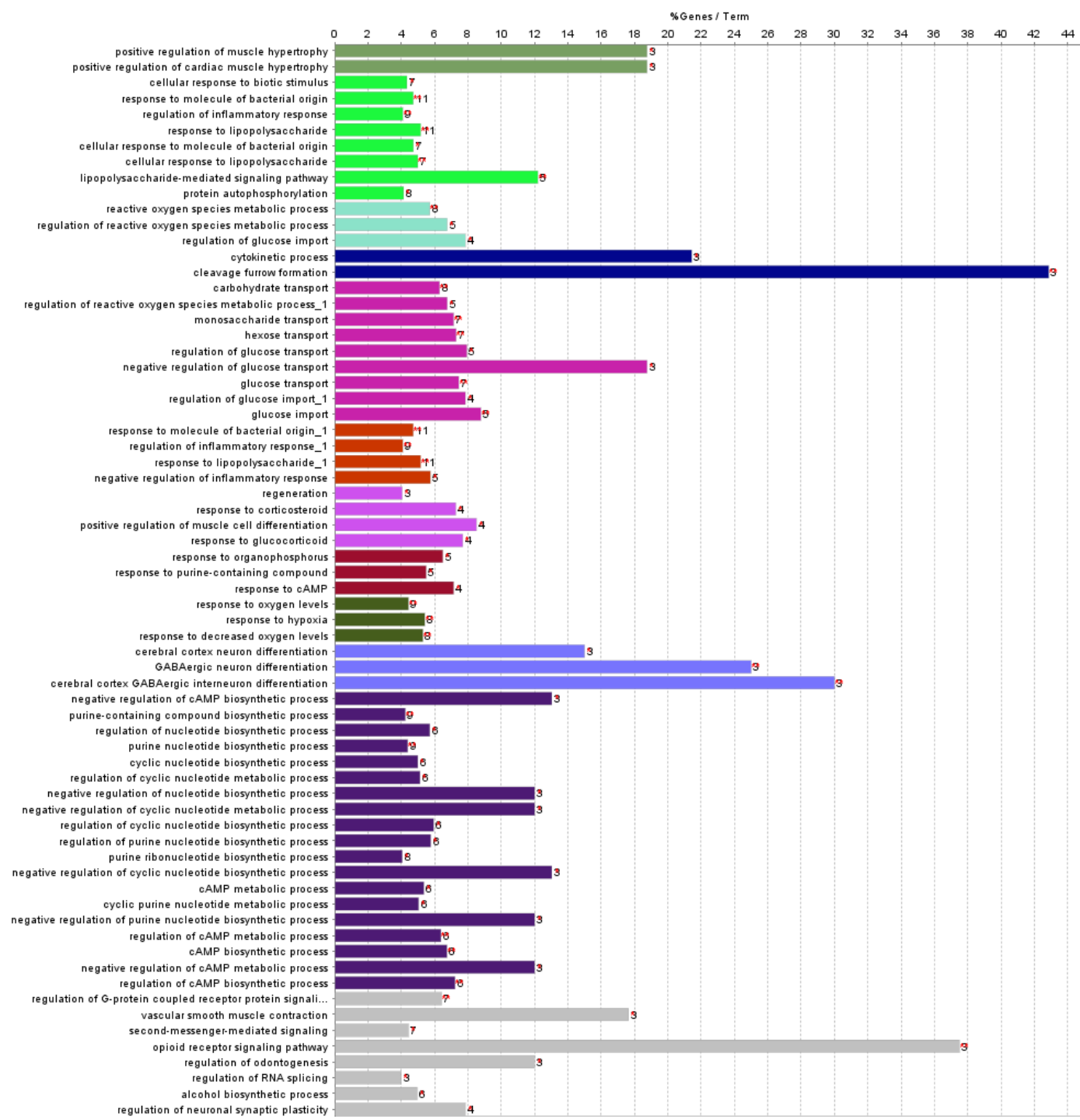

Supplement: S1 Fig — This bar-chart provides information on the functions and the number of genes for which the function was enriched at p-value < = 0.05. (PDF) [file pone.0131589.s001.pdf]
